# Review of rigid esophagoscopy in a Tertiary Hospital in Ghana

**DOI:** 10.11604/pamj.2021.39.64.25438

**Published:** 2021-05-21

**Authors:** Theophilus Adjeso, Adamu Issaka, Iddrisu Baba Yabasin

**Affiliations:** 1 Department of Ear, Nose and Throat, School of Medicine, University for Development Studies, Tamale, Ghana,; 2 Tamale Teaching Hospital (TTH), P.O. Box 16, Tamale, Ghana,; 3 Department of Surgery, School Medicine, University for Development Studies, Tamale, Ghana,; 4 Department of Anaesthesiology and Intensive Care, School of Medicine, University for Development Studies, Tamale, Ghana

**Keywords:** Esophagoscopy, coins, Ghana

## Abstract

Rigid esophagoscopy is a common endoscopic procedure worldwide for both diagnostic and therapeutic purposes. Even though this procedure is performed commonly in our center no published reports exist. We reviewed our experience with rigid esophagoscopy. This was a 9-year review of rigid esophagoscopy, done under general anaesthesia, at ENT and Cardiothoracic Units of Tamale Teaching Hospital. Parameters evaluated were patients´ demographics, indication for rigid esophagoscopy and outcome of the procedure. One hundred and fifteen cases of rigid esophagoscopies were evaluated. The ages ranged from 10 months to 87 years with a peak incidence 69.6% (n = 80) occurring within the first decade of life and a male preponderance of 54.8% (n = 63). Majority of the cases were emergencies 87.8% (n =101) and for therapeutic reasons 87% (n =100). The most common findings during esophagoscopy were: coins 60.9% (n = 70), fish bone 11.3% (n = 13), esophageal tumours 7.8% (n = 9) and dentures 5.2% (n = 6). All the cases were successfully treated with no mortality recorded. Rigid esophagoscopy was more commonly performed in males with peak age incidence occurring during the first decade of life. Emergency patients and esophagoscopy with therapeutic intent constituted the largest two groups in this study. Coins, fish bone, esophageal tumours and dentures were the most common findings. There was no mortality recorded.

## Introduction

Rigid esophagoscopy is a common surgical procedure performed by surgeons worldwide. The earliest record of this procedure dates back to the fourth century (AD) associated with sword swallowing [1]. The advancement in the technique and instrumentation is credited to the pioneering works of Adolf Kussmaul and Chevalier Jackson in the 19^th^ and 20^th^ centuries respectively [1, 2]. Esophagoscopy is used in the management of esophageal diseases with both diagnostic and therapeutic intents [3, 4]. Over the last two to three decades flexible esophagoscopy has been used as the main tool for diagnosis of esophageal conditions in place of rigid esophagoscopy. Nonetheless rigid esophagoscopy continues to be very useful in the management of esophageal diseases especially for foreign body extraction and complicated biopsies [5]. Complications of rigid esophagoscopy include esophageal tear, esophageal perforation, mediastinitis, and internal carotid artery dissection, with esophageal perforation being the most common [1, 2, 6].

Since the mid-1990s, transnasal esophagoscopy (TNE) has gained ascendancy in the treatment of esophageal conditions in advanced countries due to its advantages (no need for sedation and cost-saving, amongst others) over the more traditional esophagoscopies (rigid and flexible) [7]. Literature within the West African subregion is focused mainly on its use in the management of esophageal foreign bodies with varied foreign body pattern, occurring largely in children below the age of ten years [1, 8]. Rigid esophagoscopy is our preferred method for treating esophageal diseases especially foreign body ingestion in our hospital [1]. We have no published reports on rigid esophagoscopy from our setting. This study therefore sought to review our experience with rigid esophagoscopy in Tamale Teaching Hospital (TTH).

## Methods

This was a descriptive retrospective study of all patients managed with rigid esophagoscopy under general anaesthesia at the Ear, Nose and Throat (ENT) and Cardiothoracic Surgery Units of the Department of Surgery, TTH from January 2011 to December 2019. The data was extracted from the electronic theatre records. The parameters analyzed included the patient´s demographics, indication for esophagoscopy and outcome of procedure. The age was categorized into three age groups in decades. All the cases of rigid esophagoscopy were done under general anaesthesia using a Negus distally illuminating esophagoscope (Karl Storz). Excluded from this study were cases with incomplete information, cases managed under direct vision or with direct laryngoscopy. All extracted data were entered into a Microsoft Excel spreadsheet and cleaned for typographical errors and double entry. Cleaned data was then exported to SPSS version 20.0 for descriptive statistical analysis using means, median, frequencies and standard deviation. Ethical approval for this retrospective institutional study was obtained from the Ethical Review Board of Tamale Teaching Hospital.

## Results

One hundred and fifteen cases of rigid esophagoscopies were done during the study period. The age ranged from 10 months to 87 years with peak incidence 69.6% (n = 80) occurring within the first decade of life. The second most common age group were the above fifty years 7.8% (n = 9) with the least, 5.2% (n = 6) being within 11-50 years. There were 54.8% (n = 63) males with a male to female ratio of 1.2: 1 (Table 1).

**Table 1:** age and sex distribution of patients

Age Group (years)	Female N (%)	Male N (%)	Total N (%)
≤ 10	37 (32.2)	43(37.4)	80 (69.6)
11-50	6 (5.2)	14 (12.2)	20 (17.4)
> 50	9 (7.8)	6 (5.2)	15 (13.0)
**Total**	**52 (45.2)**	**63 (54.8)**	**115 (100)**

A majority of the rigid esophagoscopies 87.8% (n = 101) were done as emergencies with 87% (n = 100) of them being for therapeutic purposes (Table 2). Coins 60.9% (n = 70), fish bone 11.3% (n = 13), esophageal tumours 7.8% (n = 9) and dentures 5.2% (n = 6) were the most common findings during rigid esophagoscopy. Other findings during rigid esophagoscopy included achalasia, alkaline battery, cloth clip wire, esophageal stricture, earring, kola, meat/meat bone, nail head, office pin, shea and zip (Table 3). All patients were treated successfully with rigid esophagoscopy with no associated complication nor death during the study period (Table 2).

**Table 2:** procedure, intent and outcomes of rigid esophagoscopy

Variable	Frequency	Percentage (%)
**Procedure**		
Elective	14	12.2
Emergency	101	87.8
**Total**	**115**	**100**
**Intent**		
Diagnostic	15	13
Therapeutic	100	87
**Total**	**115**	**100**
**Outcomes**		
Complication	0	0
Death	0	0.0
Discharge	115	100
**Total**	**115**	**100**

**Table 3:** findings during rigid esophagoscopy

Findings	Frequency	Percentage (%)
Coins	70	60.9
Fish bone	13	11.3
Esophageal cancer	9	7.8
Denture	6	5.2
Achalasia	2	1.7
Alkaline battery	2	1.7
Esophageal stricture	2	1.7
Meat/meat bone	2	1.7
Cloth clip wire	1	0.9
Ear ring	1	0.9
Kola	1	0.9
Laryngeal tumour	1	0.9
Mediastinal mass	1	0.9
Nail head	1	0.9
Office pin	1	0.9
Shea nut	1	0.9
Unspecified (US)	1	0.9
Zip	1	0.9
**Total**	**115**	**100**

## Discussion

Rigid esophagoscopy still remains a useful and safe procedure for the management of esophageal conditions, in experienced hands, especially in resource constrained centres where flexible esophagoscopy and TNE are not routinely available. Esophagoscopy has undergone modification and advancement since its first mention in the fourth century [8]. This current study found that a majority of the cases for rigid esophagoscopy in our centre were aged ten years and below with males predominating. These findings were consistent with previous studies within the West African subregion and other parts of the world [1, 2, 8]. However, this was at variance with the study by Pino RV *et al*. who found a majority of their cases for rigid esophagoscopy were females with an average age of 68 years [9]. This difference is due to the fact that their study essentially evaluated only the adult population, whereas our study included children.

A majority of the cases of rigid esophagoscopy in our study were emergencies which were carried out for therapeutic purposes. This was found to be similar to a study done in Osun State, Nigeria, where all their cases of rigid esophagoscopy were done as emergencies [8]. However, in another study less than 15% of the cases of rigid esophagoscopy were done as emergencies [9]. This was due to the differences in the study populations. The most common findings during rigid esophagoscopy in our study were coins (60.9%, Table 2). This was similar to other published series [10-15]. In the case of head and neck tumours, rigid esophagoscopies were done as part of panendoscopic staging workup and to identify synchronous esophageal tumours, as stated in a publication by McGarey *et al*. [16]. All the patients in our study were successfully treated with rigid esophagoscopy. We did not record any complication or mortality in our study; however, complication and mortality rates following rigid esophagoscopy have been reported to be 7.2% and 1.5% respectively [17-19].

### Limitations

Our study had limitation in that we did not evaluate the presenting symptoms of the patients, the duration of foreign body ingestion and the location of the foreign bodies. In addition, the study was a retrospective review with its inherent loss of data. However, this study would form a baseline for future prospective studies from our center, Ghana and the West African subregion.

## Conclusion

Rigid esophagoscopy is a safe procedure in experienced hands. A majority of the cases were performed as emergency procedures for therapeutic purposes. The most common indications for rigid esophagoscopy were foreign body ingestion, mostly found in children who formed the majority for this study, and esophageal tumours. All the patients were treated successfully with no mortality recorded.

### What is known about this topic

- There are vast publications on rigid esophagoscopy in Africa;
- Coin is the most common finding during rigid esophagoscopy worldwide;
- Publications on rigid esophagoscopy within the Subregion is ocused mainly on esophageal foreign bodies.

### What this study adds

- Our experience with rigid esophagoscopy in Northern Ghana;
- Rigid esophagoscopy in our center for management of esophageal diseases has widened to include esophageal foreign bodies and esophageal malignancies.
